# Salivary density of *Streptococcus mutans* and *Streptococcus sobrinus* and dental caries in children and adolescents with Down syndrome

**DOI:** 10.1590/1678-7757-2016-0241

**Published:** 2017

**Authors:** Flávia SCALIONI, Camila CARRADA, Fernanda MACHADO, DEVITO Karina, Luiz Cláudio RIBEIRO, Dionéia CESAR, Rosangela RIBEIRO

**Affiliations:** 1 Universidade Federal de Juiz de Fora, Faculdade de Odontologia, Departamento de Odontopediatria, Juiz de Fora, MG, Brasil.; 2 Universidade Federal de Minas Gerais, Faculdade de Odontologia, Belo Horizonte, MG, Brasil.; 3 Faculdade Estácio de Sá, Faculdade de Odontologia, Juiz de Fora, MG, Brasil.; 4 Universidade Federal de Juiz de Fora, Faculdade de Odontologia, Departamento de Clínica Odontológica, Juiz de Fora, MG, Brasil.; 5 Universidade Federal de Juiz de Fora, Instituto de Ciências Exatas, Departamento de Estatística, Juiz de Fora, MG, Brasil.; 6 Universidade Federal de Juiz de Fora, Instituto de Ciências Biológicas, Departamento de Biologia Molecular, Juiz de Fora, MG, Brasil.

**Keywords:** Down syndrome, Dental caries, Microbiology, Bacteria, Fluorescence *in situ* hybridization

## Abstract

*Streptococcus mutans* and *Streptococcus sobrinus* are strongly associated with dental caries. However, the relationship between oral streptococci and dental caries in children with Down syndrome is not well characterized.

**Objective:**

To assess and compare dental caries experience and salivary *S. mutans*, *S. sobrinus*, and *streptococci* counts between groups of Down syndrome and non-Down syndrome children and adolescents.

**Material and Methods:**

This study included a sample of 30 Down syndrome children and adolescents (G-DS) and 30 age- and sex-matched non-Down syndrome subjects (G-ND). Dental caries experience was estimated by the number of decayed, missing, and filled teeth in the primary dentition and the permanent dentition. Unstimulated whole saliva samples were collected from all participants. The fluorescence *in situ* hybridization technique was used to identify the presence and counts of the bacteria. The statistical analysis included chi-square, Student’s t-test and Spearman’s correlation.

**Results:**

The G-DS exhibited a significantly higher caries-free rate (p<0.001) and a lower *S. mutans* salivary density (p<0.001). No significant differences were found in the salivary densities of *S. sobrinus* or *streptococci* between the groups (p=0.09 and p=0.21, respectively). The salivary *S. mutans* or *S. sobrinus* densities were not associated with dental caries experience in neither group.

**Conclusion:**

The reduced dental caries experience observed in this group of Down syndrome children and adolescents cannot be attributed to lower salivary *S. mutans* densities, as determined with the fluorescence in situ hybridization technique.

## Introduction

Down syndrome (DS) is a genetic disorder caused by a trisomy of chromosome 21 that was first described in 1866 by John Longden Hayden Down. DS is the most common chromosomal anomaly of the human species with an incidence of 1:800 to 1:1,000 births. The main clinical characteristics of DS include mental retardation and cardiovascular, haematopoietic, musculoskeletal, nervous system, and immunological system anomalies. These effects, particularly those in the immune system, result in an increased susceptibility to infection^8,12,25^.

Numerous oral abnormalities have been described in DS individuals including malformations of the small palate and maxilla, mouth breathing resulting in dry mouth, fissured tongue and lips, delayed tooth eruption, dental agenesis, low incidence of dental caries, high incidence of periodontal diseases, high incidence of mucosal ulcers, candidiasis, and acute necrotizing ulcerative gingivitis, compared with healthy individuals. Patients with DS also demonstrate macrologlossia, imbalanced occlusal and soft tissues forces, open bite, impaired chewing and consequent difficulty in self-cleansing of teeth^5,12,25^.

One of the most prominent oral manifestations in DS subjects is a low prevalence of dental caries^4,8,9,12,13^, despite exposure to risk factors, such as a cariogenic diet, decreased salivary flow, mouth breathing, unbalanced occlusal forces, and poor access to oral hygiene^25,28^. Some studies have addressed the etiology of this low prevalence of dental caries, but the exact mechanism remains unclear. Some of the hypotheses suggested to explain the low prevalence of dental caries include the following: delayed tooth eruption in combination with an altered chronology of eruption; the high frequency of hypodontia; differences in the composition, pH, and buffering capacity of the saliva and the salivary flow^8,9,25^, and differences in the cariogenic microbiota^7,9,28^.

Microbial diversity comprises the number of species present (species richness) and the number of individuals of each species (uniformity). The knowledge about microbial diversity is important, given that a microbial community may change in terms of the number of individuals *per* species in response to changing conditions that favor their growth^27^.

*Streptococcus mutans* and *Streptococcus sobrinus* are strongly associated with dental caries. However, the relationship between oral streptococci and dental caries in children with DS is not well characterized. While some studies have shown that the occurrence of dental caries is associated with *S. mutans* counts in children and adolescents with DS^2,9,20,28^, other authors have not found such association^7^. Thus, investigations to clarify the ecologies of the oral cavities of individuals with DS are necessary^7^.

The aforementioned ecologies could best be elucidated with molecular methods, which are rarely used in studies with this population group^9^. Fluorescence *in situ* hybridization (FISH) technique allows for the visualization, differentiation, and quantification of various oral bacterial species^1,3^ because it combines the accuracy of molecular genetics with the visual information of microscopy^22,23^. Therefore, this study aimed to assess the salivary densities of *S. mutans*, *S. sobrinus*, and *streptococci* and dental caries experience, in a group of DS children and adolescents.

## Material and Methods

### Study design and sample characteristics

This cross-sectional study was approved by the Human Research Ethics Committee (registry no. 383/2011) of the University Hospital, Federal University of Juiz de Fora, and written informed consent was obtained from the parents. Additionally, the participants were required to agree with the dental examination and saliva sampling.

Thirty DS children and adolescents with karyotype-confirmed diagnoses, who were attended by the Association of Parents and Friends of the Exceptional, formed the DS group (G-DS); 30 children and adolescents without DS formed the non-Down group (G-ND). They were selected from individuals in the same age group who were enrolled in an educational institution in the city of Juiz de Fora, Minas Gerais, Brazil. All DS children lived at home with their parents and all attended a part-time school for disabled children. Members of the control group were selected at random from one of the public schools in Juiz de Fora. All children in the control group also lived at home with their parents.

The study was planned with a total of 30 children and adolescents in G-DS and 30 in G-ND, considering a significance level of 0.05, standard deviation of 1, and difference between mean density of *S. mutans* species of 1.4. The power of the Student’s *t*-test for independent samples was 0.999.

For inclusion in this study, the participants with and without DS had to be between the ages of 3 and 12 years and exhibit primary or mixed dentition. To calculate the ages, the most recent birthdays were considered. No children or adolescents with systemic diseases other than DS, undergoing orthodontic treatment, and/or using antimicrobial drugs in the previous three weeks were included. Only children and adolescents who were able to cooperate for collection of saliva were included in the study.

### Dental caries experience

Prior to the beginning of the study, a single dental examiner (FARS) was trained by a specialist in pediatric dentistry (i.e., the gold standard – RAR) to ensure consistency in the dental caries diagnoses. Six non-Down syndrome children (10% of the total sample) were previously examined twice, with intervals of 7 days. The Cohen’s Kappa coefficients were *ĸ*=0.94 for the intra-examiner agreement and *ĸ*=0.92 for the inter-examiner agreement. The dental examinations were performed under artificial light with the participants seated in a dental chair.

Dental caries experience was estimated based on the presence of decayed, missing, or filled primary and permanent teeth, i.e., the dmft and DMFT indexes, which were scored according to the criteria of the World Health Organization^31^. The examinations were performed with an exploratory probe and a dental mirror under artificial light, after drying the dental elements with compressed air. All necessary instruments were sterilized before the examinations.

### Detection, identification, and quantification of cariogenic oral bacteria

Whole unstimulated saliva samples were collected from the floor of the mouth of each volunteer, using a disposable plastic Pasteur pipette (Qingdao AMA Co., Ltd., Qingdao, Shandong, China) in a clinic room. Samples were collected following a clinical examination between 8:00 am and 12:00 pm^5,12^, and at least one hour after eating, brushing the teeth, or rinsing the mouth^12^.

After collection, 180 µL of the saliva were transferred, with the aid of an automatic pipette, into a microcentrifuge tube containing 20 µL of 20% paraformaldehyde. The samples were maintained under refrigeration and transported immediately to the Laboratory of Ecology and Molecular Biology of Microorganisms at the Federal University of Juiz de Fora. The fixed samples were stored at -20°C for microbiological analysis.

**Figure 1:**
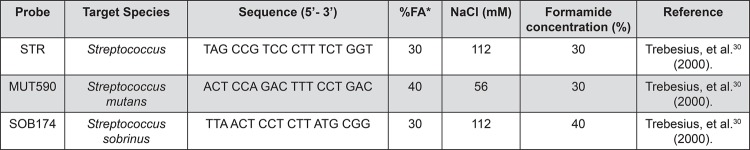
Oligonucleotide probes, 16S rRNA (Operon Techbologies®) marked with Cy3 fluorochrome for the identification of oral microorganisms

The identification and quantification of bacteria were determined by the FISH technique. The samples were fixed in 20% paraformaldehyde (2% final concentration) and filtered through a 0.22 μm Millipore polycarbonate white filter. Oligonucleotide probes, 16S rRNA (Operon Technologies Inc., USA) were used, marked with Cy3 fluorochrome (Indo-carbocyanine) to identify the oral microorganisms (Figure 1).

*Percentage of formamide (FA) in hybridization solution

Subsequently, the samples were stained with 4’,6-diamidino-2-phenylindole (DAPI) to quantify the total bacterial density. A negative control marker (5’3CCTAGTGACGCCGTCGA – 3’) with no specificity for any bacterial group and some positive control markers, EUBI (GCT GCC TCC CGT AGG AGT), EUBII (GCA GCC ACC CGT AGG TGT), and EUBIII (GCT GCC ACC CGT AGG TGT), were used to evaluate hybridization efficiency^11^. The filters were then divided into seven parts, i.e., one for each specific probe, including the positive probe and the negative probe. Each piece of the filter was placed on a glass slide covered with parafilm and covered with 30 µL of hybridization solution with a final concentration of 2.5 ng/µL of the oligonucleotide probe. The hybridization solution was composed of 0.9 M NaCl, 20 mM Tris-HCl (pH 7.4), 0.01% sodium dodecyl sulphate, and a concentration of formamide that was specific for each bacterium. The sample was incubated in a heater at 42°C overnight. After hybridization, the sample was transferred to a washing solution containing 20 mM Tris-HCl (pH 7.4), 5 mM EDTA, 0.01% sodium dodecyl sulphate, and a NaCl concentration suitable for the specific probe. The sample was then incubated at 48°C for 15 minutes. The bacterial cells were stained with 2 µg of DAPI *per* mL so that the total bacterial density could be acquired. Each piece of the filter was immersed in 80% ethanol (v/v) three times and dried. Finally, the slide was mounted using glycerol and Vecta shield (Vector Laboratories Inc., Burlingame, California, The United States of America) at a ratio of 4:1.

The total bacterial cells of each species were counted using an Olympus BX60 epifluorescence microscope equipped with the 41007a filter for the Cy3 marker, and a 31000 filter for the DAPI at a magnification of 1000x. The counting was performed in ten random fields by a single researcher (FARS) who had been trained by an experienced researcher (DEC). The final number of bacteria was calculated by multiplying the dilutions made during sample treatment. The percentages of each species in relation to the total bacterial cell counts were calculated. The results are expressed in cells/mL.

### Statistical analysis

The data were organized into a database using the Statistical Package for Social Sciences (SPSS), version 15.0 for Windows. Categorical variables are described as frequency distributions. Descriptive measures (i.e., mean, standard deviation, and minimum and maximum values) were used to describe the continuous variables related to the investigated bacteria. The chi-square test was used to analyze age, sex, previous dental experience, and dental caries experience variables. The Student’s *t*-test was used to compare the salivary densities of the tested bacteria between the groups. Spearman’s correlation test was used to assess the associations between the salivary counts of *S. mutans* and *S. sobrinus* and the dental caries experience. The significance level was set at 5%.

## Results

The total sample included 60 children and adolescents between 3 and 12 years of age who resided in the city of Juiz de Fora. Table 1 shows characterizations of the sample according to age, sex, and previous dental experience. Among the participants in the G-DS, 53.3% (16/30) had at least one previous dental experience. In the G-ND, the majority of the participants (90.00%, 27/30) had already visited a dentist. This difference was statistically significant (*p*=0.003).

**Table 1:** Sample characteristics in relation to age, sex, and previous dental experience

Variables	Total sample (N=60)	G-DS (N=30)	G-ND (N=30)	p value
Age (years)				
Mean±standard-deviation (years)	6.95±2.38	6.37±2.50	7.53±2.15	0.057ns
Range (years)	3/12	3/12	4/12	
Sex (n / %)				
Male (n / %) Female (n / %)	31/51.70 29/48.30	17/56.70 13/43.30	14/43.70 16/53.30	0.606ns
Previous dental experience (n / %)				
Yes (n / %) No (n / %)	43/71.70 17/28.30	16/53.30 14/46.70	27/90.00 3/10.00	0.003

ns – Qui-square test.

Table 2 shows the data on the clinical examination regarding dental caries experience in the G-DS and G-ND. The participants were divided into a group with a dmft or DMFT=0 (considered free of caries) and a group with a dmft or DMFT≥1. The G-DS had a higher percentage of caries-free children and adolescents than the G-ND (66.66% – 20/30 children versus 3.33% – 1/30 children; *p*<0.001).

**Table 2:** Comparison of the caries experience data between the two groups (G-DS and G-ND)

Variable	Total sample (N=60)		G-DS (N=30)		G-ND (N= 30)		p value
dmf-t or DMF-T	N	%	n	%	N	%	p<0.001*
= 0	21	35	20	66.70	1	3.30	
≥ 1	39	65	10	33.3	29	96.70	

* Significant difference – Qui-square test.

Table 3 presents the descriptive measures of the observed bacterial densities in the saliva samples (cells/mLx10^8^). The Student’s *t*-test revealed a significantly lower mean density of *S. mutans* in the DS group (*p*<0.001).

**Table 3:** Descriptive measures of the bacterial densities (cells/mLX 108) in the saliva samples and results of the comparison between groups

Bacteria	Mean (SD)		Minimum		Maximum		p value
	G-DS	G-ND	G-DS	G-ND	G-DS	G-ND	
*S. mutans*	0.397 (0.532)	1.833 (2.255)	0.00	0.00	2.37	8.18	<0.001*
*S. sobrinus*	1.623 (0.858)	1.193 (1,623)	0.00	0.00	2.91	5.44	0.09ns
*Streptococci*	1.898 (5.116)	9.690 (5.770)	0.00	0.01	28.14	22.24	0.21ns
DAPI	55.711 (32.046)	60.753 (27.879)	15.98	13.94	131.97	158.32	0.52ns

* Significant difference –Student’s t-test.

ns – Student’s t-test.

The densities of *S. mutans* and *S. sobrinus* in the saliva samples (cells/mL x 10^8^) of each group of children and adolescents were also compared according to dental caries experience. Student’s *t*-tests revealed no significant differences in the density of either bacterium (Table 4). Spearman’s correlation tests revealed that the salivary densities of *S. mutans* and *S. sobrinus* were not associated with the dental caries experience of either group (*S. mutans*: G-DS, *p*=0.931; G-ND, *p*=0.462; *S. sobrinus*: G-DS, *p*=0.697; G-ND, *p*=0.230).

**Table 4:** Intra-group comparisons of the densities of S. mutans and S. sobrinus in the saliva samples (cells/mLX 108) between groups according to caries experience

Bacteria	G-DS (N=30)			G-ND (N=30)		
	dmft or DMFT=0	dmft or DMFT≥1	p value	dmft or DMFT=0	dmft or DMFT≥1	p value
*S. mutans*	0.4005	0.3910	0.964	23.200	18.159	0.830ns
*S. sobrinus*	0.6715	0.4970	0.608	24.100	11.514	0.455ns

ns – Student’s t-test.

## Discussion

This study was conducted to evaluate and compare the salivary densities of *S. mutans*, *S. sobrinus*, and *Streptococcus,* as well as the dental caries experience among a convenience sample of children and adolescents with and without Down syndrome. The groups exhibited similar distributions of age, gender, and origin. The understanding and cooperation of the participants were part of the inclusion criteria because clinical examinations and saliva collections can be particularly difficult for some children with DS and severe intellectual disabilities. The internal validity of the study was ensured by the intra- and inter-examiner calibrations. In addition, a high power for independent samples was obtained.

The method used to detect dental caries was based on clinical examinations of the children using a dental mirror and an exploratory probe to record the DMFT and dmft values according to the criteria of the World Health Organization^31^. This method is objective and efficient for detecting dental caries^4,10,11,19,26^.

The results of dental caries experience revealed a significantly higher frequency of caries-free children and adolescents in the G-DS, which is consistent with results reported in previous investigations^4,6,9-11,23,25,28^. We could suggest that the highly significant difference in the frequencies of caries-free children and adolescents between the two groups resulted from the sample composition of the G-ND. Because dental caries are strongly correlated with dietary habits, oral hygiene, and familial predisposition^5^, the ideal control group would have included siblings of the children with DS who were matched in terms of sex and age. However, the participants in the G-DS either had no siblings or had siblings over 18 years of age.

Differences in cariogenic microbiota could explain the low prevalence of dental caries that has frequently been observed in children with DS. However, the role of cariogenic bacteria in the etiology of dental caries in Down syndrome children is not entirely clear. The conflicting results of previous studies may be attributed to differences in the methods used. Very few studies have employed molecular methods to differentiate oral cariogenic bacteria in DS subjects^9^. To our knowledge, this is the first study to use the FISH technique to detect and quantify cariogenic bacteria in the saliva of DS children and adolescents. The FISH technique provides direct quantitative results, does not require prior culturing, allows for the visualization and counting of individual microbial cells via microscopy, and is a rapid and objective method^14,18,22^. Moreover, the FISH technique has been proven effective in the detection of *S. mutans* and *S. sobrinus* ^14,18,24,29^.

To detect and quantify cariogenic oral bacteria, unstimulated saliva samples were collected^12,20^. Saliva represents an easy and non-invasive means for obtaining bacterial samples from all of the oral sites^16^. The samples were collected preferentially in the morning, to minimize the effects of circadian rhythms^5^.

In the total sample, *S. mutans*, *S. sobrinus*, and streptococci were present in 85% (51/60), 87% (52/60), and 93% (56/60) of the participants, respectively. In the G-DS, *S. mutans*, *S. sobrinus*, and streptococci were present in 76% (23/30), 82% (24/30), and 88% (26/30) of the participants, respectively. In the G-ND, the frequencies were 94% (28/30) for *S. mutans* and *S. sobrinus,* and 100% (30/30) for streptococci (data not shown). These results showed a high prevalence of cariogenic bacteria that was likely due to greater sensitivity of the FISH technique compared with conventional culture methods.

The comparisons of the mean bacterial densities of the two groups revealed that the children and adolescents with DS had lower density of *S. mutans* (*p*<0.05), higher density of *S. sobrinus* (*p*<0.10), and lower density of streptococci (*p*>0.20). The *S. mutans*’ results corroborate the findings of previous studies^7,11,18^. However, other studies have reported similar^9^ or higher counts^17^. No comparisons with previous results could be made regarding *S. sobrinus* because the studies that have evaluated the presence of this species did not include control groups of non-Down children^7^ or did not separately present the results for this species^5,17^. The prevalence of *S. sobrinus* is more strongly associated with future dental caries activity, particularly on the smooth surfaces^17^. Further studies are required to demonstrate the influence of salivary densities of *S. mutans* and *S. sobrinus* on the incidence of dental caries in children and adolescents with DS.

Comparisons of the mean bacterial densities within the G-DS revealed no differences between the participants with and without caries for any of the investigated bacteria. These results contrast with previous observations of correlations between *S. mutans* density and caries experience in children and adolescents with DS^2,9,20,28^, but agree with the data obtained by other authors^9^. Notably, the low prevalence of dental caries among children with DS reported in a study by Cogulu, et al.^9^ (2006) was attributed to colonization by *S. mutans* strains with less cariogenic profiles, or the presence of different acidogenic or aciduric strains. Additionally, one previous study reported inverse relationships between periodontal disease and dental caries, both in terms of clinical and microbiological findings; specifically, this study found an inverse correlation between *Porphyromonas gingivalis* and *S. mutans* ^29^. The results of a study in development that aims to assess the salivary densities of periodontal pathogens by means of the FISH technique, in the same sample of DS children and adolescents, may confirm this inverse relationship.

Our results showed that children and adolescents with Down syndrome have less extensive caries experience. The FISH technique revealed that children and adolescents with DS have significantly lower salivary density of *S. mutans*. Not only should the prevalence be considered, the number of individuals of each species is important in determining cariogenic processes. Microorganisms are capable of expressing a gene according to the cell-to-cell density of individuals, called quorum sensing^21^. The FISH technique allows for determining the prevalence and the quantification of individuals of the species selected from the study. Variability among individuals should be considered, which can be observed by the high standard deviation values. However, the *S. mutans* density did not differ between the DS children and adolescents, with and without caries experience; therefore, it is not possible to ascribe the lower dental caries experience to the lower number of this species in the group with Down syndrome.

Notwithstanding, it should be noted that the frequency of caries-free children and adolescents in the G-DS was still below the goals proposed by the WHO for 2010^15^. Strategies to achieve these goals should be implemented and should involve the teams of professionals who take care of children and adolescents with Down syndrome. Moreover, these professionals should recognize the important role of Dentistry in achieving a higher quality of life for this portion of the population.

## Conclusions

In conclusion, we found that DS children and adolescents present a lower dental caries experience and a lower salivary density of *S. mutans* than non-Down controls. However, the reduced dental caries experience observed in this group of Down syndrome subjects could not be attributed to the lower salivary *S. mutans* density, as determined by the fluorescence *in situ* hybridization technique.
